# Religiosity and alcohol use in adolescents with orofacial cleft: correlational study

**DOI:** 10.1590/1984-0462/2025/43/2023265

**Published:** 2024-10-04

**Authors:** Lázaro Clarindo Celestino, Ana Paula Fukushiro, Flávia Maria Ravagnani Neves Cintra, Gesiane Cristina Bom, Claudia Regina Matiole, Armando dos Santos Trettene

**Affiliations:** a Universidade de São Paulo, Hospital de Reabilitação de Anomalias Craniofaciais, Bauru, SP, Brazil.

**Keywords:** Religion and medicine, Teens, Alcohol, Cleft palate, Cleft lip, Religião e medicina, Adolescentes, Álcool, Fissura palatina, Fenda labial

## Abstract

**Objective:**

To evaluate the correlation between religiosity and alcohol use among adolescents with orofacial clefts.

**Methods:**

Cross-sectional study, developed in a Brazilian public and tertiary hospital, between December 2021 and March 2022. Data collection was hybrid, and three instruments were used: Sociodemographic Questionnaire, Durel Religiosity Scale, and the Alcohol Use Disorders Identification Test. For statistical analysis, the following tests were used: χ^2^, Fisher’s Exact, Mann-Whitney and Spearman’s Correlation Coefficient, in addition to analyses of linear correlation strength and bivariate logistic regression. The significance level adopted for all tests was 5% (p≤0.05).

**Results:**

370 adolescents participated, with a mean age of 15.2 years (±1.8). Among them, 23 (5.4%) used alcohol riskly or harmfully, being more frequent among male adolescents (p=0.001), those of mixed race (p=0.046), attending high school (p=0.011), with no religion (p<0.001), or who did not attend religious services (p<0.001). Levels of organizational, non-organizational and intrinsic religiosity were significantly lower among adolescents with risky or harmful alcohol use (p=0.005; p<0.001 and p=0.002, respectively). There was a moderate correlation between risky or harmful alcohol use and non-organizational (r=0.31; p=0.002) and intrinsic (r=0.36; p<0.001) religiosity. Male adolescents (p<0.001; OR=6.58), closest in age to 18 years (p<0.001; OR=1.37), and non-practitioners of religion (p<0.001; OR=6. 48) presented higher odds of risky or harmful alcohol use.

**Conclusions:**

Adolescents with higher levels of organizational and intrinsic religiosity used less alcohol, while males, closest in age to 18 years, and non-practitioners of religion presented higher odds of using alcohol riskly or harmfully.

## RESUMO

**Objetivo:**

Avaliar a correlação entre a religiosidade e o uso de álcool em adolescentes com fissura orofacial.

**Métodos:**

Estudo transversal, desenvolvido em um hospital público e terciário brasileiro entre dezembro de 2021 e março de 2022. A coleta de dados foi híbrida, e foram utilizados três instrumentos: Questionário Sociodemográfico, Escala de Religiosidade de Durel e o *Alcohol Use Disorders Identification Test.* Para a análise estatística foram empregados os testes: qui-quadrado, exato de Fisher, Mann-Whitney e o coeficiente de correlação de Spearman, além das análises de forças de correlação linear e de regressão logística bivariada. O nível de significância adotado para todos os testes foi de 5% (p≤0,05).

**Resultados:**

Participaram 370 adolescentes, com média de idade de 15,2 anos (±1,8). Entre eles, 23 (5,4%) usavam o álcool de forma arriscada ou prejudicial. A religiosidade organizacional, não organizacional e a intrínseca foram significativamente menos frequentes entre adolescentes que utilizavam o álcool de forma arriscada ou prejudicial (p=0,005; p<0,001 e p=0,002, respectivamente). Evidenciou-se correlação moderada entre o uso arriscado ou prejudicial do álcool e a religiosidade não organizacional (r=0,31; p=0,002) e a intrínseca (r=0,36; p<0,001). Apresentaram maiores chances de usar o álcool adolescentes do sexo masculino (p<0,001; *odds ratio* — OR=6,58), com idade mais próxima aos 18 anos (p<0,001; OR=1,37) e que não praticavam a religião (p<0,001; OR=6,48).

**Conclusões:**

Adolescentes com maiores níveis de religiosidade organizacional e intrínseca utilizaram menos frequentemente álcool de forma arriscada ou prejudicial, enquanto ser homem, possuir idade mais próxima aos 18 anos e não praticar a religião aumentou a chance de utilizar álcool.

## INTRODUCTION

Patients affected by this malformation may present functional, aesthetic and psychosocial problems. Although at birth and in childhood the implications are predominantly related to functional aspects, such as nutrition, in adolescence the psychosocial aspects are highlighted, including those related to dental and hearing disorders, physical appearance, communication and socialization problems, including negative repercussions on the quality of life.^1,2,3^

Adolescents with orofacial clefts can experience prejudice and discrimination, which can result into low social acceptance and isolation, although the establishment of these behaviors depends on individual and collective demands, such as life history, family relationships, development of their rehabilitation process, cultural, religious and social standards.^4,5,6,7,8^

Therefore, it is considered that adolescents with orofacial clefts present more social vulnerability, which may favor the establishment of risk behaviors or the acquisition of harmful habits, such as the use of legal and illicit drugs,^5,6,7,8^ including alcohol. Worldwide, alcohol is recognized as the preferred substance among adolescents, representing an important public health problem and one of the main behaviors of this population.^9,10,11^ In Brazil, 26.8% of young people aged between 15 and 19 reported consuming alcohol.^12^

From this, numerous consequences are evident depending on use and abuse, including aggressive behavior, risk of accidents, abusive and unprotected sexual practice, anxiety, anguish, depression, loneliness, feelings of worthlessness, difficulty for concentrating, fatigue, risk of suicide, among others.^9,10,11^

From this perspective, religiosity stands out, as its influence encompasses the field of health, interpersonal, sociocultural relationships and the individual’s intrapsychic, where beliefs, values, behaviors and emotions gain notoriety.^11,13,14,15,16,17^

Although the benefits of religiosity as a protective factor against alcohol use and abuse among adolescents are known,^14,16^ there are no studies addressing these variables among adolescents with orofacial clefts, and our hypothesis is that religiosity acts as a protective factor against risky or harmful alcohol use among those adolescents.

In this sense, we sought to evaluate the correlation between religiosity and risky or harmful alcohol use among adolescents with orofacial clefts.

## METHOD

Cross-sectional study, guided by the reporting of observational studies in epidemiology — STROBE, carried out between December 2021 and March 2022, in a public and tertiary hospital, located in the interior of the State of São Paulo, in Brazil. This institution is a reference in the care of patients with orofacial clefts and related syndromes, being managed with resources from the Unified Health System and the São Paulo University. It has 91 beds and comprises care, teaching and research areas, being considered as an (inter)national reference.

Adolescents with orofacial clefts, of lip, palate or lip and palate, isolated, previously operated, aged between 12 and 18 years old, participated. The age range is in accordance with the the provisions of the Brazilian Child and Adolescent Statute. Adolescents with other malformations, syndromic ones, and those with clinical comorbidities, including neurological, cognitive, cardiovascular and endocrinological problems, were excluded.

For the sample calculation, an expected amount of alcohol use of 67%^18^ and error of 5% were considered. Thus, a minimum sample of 340 adolescents was estimated. Ultimately, 370 teenagers participated.

The dependent variable consisted of alcohol use, including low risk, risky use, harmful use and addiction. As independent variables, the following were included: religiosity in its three dimensions (organizational, non-organizational and intrinsic), age, gender, race, education, religion, practice of religion, socioeconomic classification, type of cleft, number of children, activity salary and marital or emotional status.

Three questionnaires were used to collect data: Sociodemographic Questionnaire, the Durel Religiosity Scale and the Alcohol Use Disorders Identification Test — AUDIT.^19,20^

To characterize the participants, a Sociodemographic Questionnaire, created by the researchers, was employed for the present study. For the socioeconomic classification, the protocol of the institution was used, encompassing the following indicators: economic situation of the family, family composition, level of education and occupation, housing condition and situation, among others, organized in a scoring system resulting into six stratifications: low, lower low, upper low, mid, lower mid, upper mid and high.^5^

The Durel Religiosity Scale, translated and validated to the Brazilian reality, was used to assess religiosity. The validation study showed adequate internal consistency (Cronbach’s α>0.80) and test-retest reliability (Intraclass Correlation Coefficient>0.90). In summary, the authors recommended its use to investigate the dimensions of religiosity in Brazilian samples, with different sociodemographic characteristics.^21^

It consists of five items divided into three domains: organizational religiosity (OR), non-organizational religiosity (NOR) and intrinsic religiosity (IR).^19^ The fact that it is an instrument with few items implies some benefits, which includes its optimization, especially when considering the participation of adolescents who, at times, are not very compliant with researches that apply extensive and complex questionnaires. However, this specificity does not impair the analysis of the constructs referring to the assessment of religiosity in its different dimensions, as it presented a high positive correlation with spirituality, religiosity and personal beliefs, being recommended for adolescents, including those with different sociodemographic characteristics.^22^

OR refers to attendance at churches and/or temples with social interaction, the score varying from one to six. The NOR is independent of other people, that is, it refers to individual religious activity (prayer, meditation), and has a score from 1 to 6. IR, as far as it is concerned, evaluates the behavior and influence that religion provides to an individual’s life, consisting of a score ranging from 3 to 15. To calculate the instrument score, it is recommended that the three domains be analyzed separately, that is, they should not be added together into a final score. The lower the value obtained, the higher the religiosity assessed.^19^

To investigate the use of alcohol, the AUDIT was used, an instrument developed by the World Health Organization, which was translated and validated for the Brazilian reality. It is self-administered and has 10 objective questions, whose score varies from 0 to 40 points and helps to identify four consumption patterns: low-risk use (0 to 7 points); risky use (8 to 15 points); harmful use (16 to 19 points), and probable dependence (20 or more points).^20^

Aiming to minimize possible biases, a pilot study was initially carried out with 20 participants, who were not included in the sample, and whose data collection was in person. Finally, no methodological changes were necessary, as well as adaptations to the data collection instruments.

Data collection was hybrid, that is, adolescents who were hospitalized or receiving outpatient care were initially invited to participate in the research. For them, data collection was carried out in a private environment, individually, which lasted, on average, 20 minutes. Thus, the objective of the research and the data collection instruments were presented and answered immediately.

Subsequently, due to the Corona Virus Disease Pandemic (COVID-19), where face-to-face services were interrupted, eligible adolescents were identified through the institution’s electronic file, with their respective telephone and email contacts. Patients attended between January 2018 and December 2020 were included. For them, the invitation to participate was made via telephone. After acceptance, a link to access the Terms of Consent/Assent and data collection instruments was sent via telephone or email, at the participant’s discretion, using the Google Forms platform.

Regardless of the methodology used for data collection, the variable “socioeconomic classification” was obtained from the medical records.

In the analysis of categorical variables, the Chi-Square Test and the Fisher’s Exact Test were used. In cases where statistical significance was found, the analysis of corrected adjusted residuals was applied. For continuous quantitative variables, the Mann-Whitney Test and the Spearman Correlation Coefficient were used, besides the analysis of linear correlation, which determines that correlation values lower than 0.30 indicate weak correlation, that is, even when statistically significant, they do not present clinical relevance; values between 0.30 and 0.50 show moderate correlation, and those above 0.50 show strong correlation.^23^

Finally, for the independent variables identified as significant, the binary logistic regression model was applied. For significant variables, identified by using the Forward LR method, Odds Ratios (OR) estimates and their respective 95% confidence intervals were obtained. The significance level adopted for all tests was 5% (p≤0.05).

The study was approved by the institution’s Research Ethics Committee, and all ethical patterns required were observed.

## RESULTS

A total of 370 adolescents participated: 168 in person and 202 online. The average age was 15.2 years (±1.8). Those with cleft lip and palate prevailed (44.05%; n=163), male (52.70%; n=195), mixed race (60.54%; n=224), with high school education (52.43%; n=194), students from public schools (92.43%; n=342), single (88.92%; n=329), from low socioeconomic class (43.24%; n=160), with their own housing (87.03%; n=322), with religion (91.35%; n=338), Catholics (47.70%; n=176), religious practitioners (72.70%; n =269), with no children (99.46%; n=368) and under unpaid work (94.59%; n=350). Among them, 23 (5.40%) used alcohol riskly or harmfully, with prevailing risky use (n=20; 5.40%) compared to harmful use (n=3; 0.80%), which, for analysis purposes, were considered jointly.

Risky or harmful alcohol use was significantly more frequent among male adolescents (p=0.001), those of mixed race (p=0.046), attending high school (p=0.011), with no religion (p<0.001), or non-practitioners of religion (p<0.001) (Table 1).

**Table 1:** Distribution of participants (n=370), regarding alcohol use according to the variables: type of cleft, gender, race, education, type of school, marital/affective status, socioeconomic classification, housing, religion, practice of religion, children, paid work. Bauru (SP), Brazil, 2022.

Variables	No or low-risk use n (%)	Risky/harmful use n (%)	p-value
Type of cleft*			
Lip	87 (23.51)	6 (1.62)	0.320
Lip and palate	150 (40.54)	13 (3.51)	
Palate	110 (29.73)	4 (1.08)	
Gender*			
Female	172 (46.49)	3 (0.81)	<0.001^†^
Male	175 (47.30)	20 (5.41)	
Race/color*			
White	93 (25.14)	4 (1.08)	0,046^†^
Brown	212 (57.30)	12 (3.24)	
Black	36 (9.73)	7 (1.89)	
Others	6 (1.62)	0 (0)	
Education*			
Elementary	169 (45.68)	4 (1.08)	0.011^†^
High school	175 (47.30)	19 (5.14)	
Technical education	3 (0.81)	0 (0)	
School*			
Private	28 (7.57)	0 (0)	0.239
Public	319 (86.22)	23 (6.22)	
Marital/affective status^‡^			
Steady union	2 (0.54)	0 (0)	0.373
Dating	35 (9.46)	4 (1.08)	
Single	310 (83.78)	19 (5.14)	
Socioeconomic status^‡^			
Low	149 (40.27)	11 (2.97)	0.107
Lower low	34 (9.19)	6 (1.62)	
Upper low	48 (12.97)	0 (0)	
Mid	74 (20.00)	4 (1.08)	
Lower mid	34 (9.19)	2 (0.54)	
Upper mid	8 (2.16)	0 (0)	
Housing*			
Own	303 (81.89)	0 (0)	0.519
Rented	44 (11.89)	19 (5.14)	
Religion*			
Catholic	162 (43.78)	14 (3.78)	<0.001^†^
Protestant	134 (36.22)	1 (0.27)	
Others	26 (7.03)	1 (0.27)	
None	25 (6.76)	7 (1.89)	
Practitioner of religion*			
No	84 (22.70)	17 (4.59)	<0.001^†^
Yes	263 (71.08)	6 (1.62)	
Children*			
No	345 (93.24)	23 (6.22)	1.000
Yes	2 (0.54)	0 (0)	
Employment bond*			
No	329 (88.92)	21 (5.68)	0.625
Yes	18 (4.86)	2 (0.54)	

*χ^2^ test;

^†^5% significance level (p≤0.05);

^‡^Fisher’s exact test.

Religiosity, in all dimensions, that is, OR, NOR and IR, were significantly less frequent among adolescents with risky or harmful alcohol use (p=0.005; p<0.001 and p=0.002, respectively) (Table 2).

**Table 2:** Distribution of participants (n=370) regarding Organizational, Non-Organizational and Intrinsic Religiosity. Bauru (SP), Brazil, 2022.

Religiosity*	Alcohol use*	n	Median	1^st^ Quartile	3^rd^ Quartile	Mean	Standard deviation	p-value
Organizational	No/low risk	347	3	2	4	3.3	1.3	0.005^†^
	Risky/harmful	23	4	3	5	4.2	1.5	
Non-organizational	No/low risk	347	3	2	5	3.5	1.5	<0.001^†^
	Risky/harmful	23	5	3	6	4.7	1.5	
Intrinsic	No/low risk	347	4	3	6	5.1	3.0	0.002^†^
	Risky/harmful	23	9	3	11	8.1	4.5	

*Mann-Whitney test;

^†^5% significance level (p≤0.05).

However, there was only a moderate correlation between risky or harmful alcohol use and NOR (r=0.31; p=0.002), and with IR (r=0.36; p<0.001), that is, the lower the NOR and IR levels, the more frequent risky or harmful alcohol use (Table 3).

**Table 3:** Correlation between Organizational, Non-Organizational, Intrinsic Religiosity, age and alcohol use among adolescents with orofacial clefts. Bauru (SP), Brazil, 2022.

Correlation variables	r*	Correlation	p-value
Alcohol use and Organizational Religiosity	0.24^†^	Weak	0.010^‡^
Alcohol use and Non-Organizational Religiosity	0.31^†^	Moderate	0.002^‡^
Alcohol use and Intrinsic Religiosity	0.36^†^	Moderate	<0.001^‡^
Alcohol use and age	0.29^†^	Weak	<0.001^‡^

*correlation value;

^†^Sperman correlation test;

^‡^5% significance level (p≤0.05).

The odds of risky or harmful alcohol use were 6.58 times higher among male adolescents, 1.37 times higher among adolescents closer to 18 years of age, and 6.48 times higher among non-practitioners of religion (Table 4).

**Table 4:** Factors associated with alcohol use among adolescents with orofacial clefts. Bauru (SP), Brazil, 2022.

Variables	No/Low risk use n (%)	Risky/harmful use n (%)	p-value	OR	95%CI
Age	14.96±2.07	16.44±1.61	<0.001*	1.37	1.05–1.78
Gender					
Female	172 (46.49)	3 (0.81)	<0.001*	6.58	1.86–23.40
Male	175 (47.30)	20 (5.41)			
Practitioner of religion					
No	84 (22.70)	17 (4.59)	<0.001*	6.48	2.38–17.55
Yes	263 (71.08)	6 (1.62)			

*5% significance level (p<0.05).

OR: odds ratio; CI: confidence interval.

## DISCUSSION

The prevalence of risky or harmful alcohol use among adolescents with orofacial clefts was 5.6%, therefore, lower than observed in other studies. Investigations carried out in Brazil with adolescents without orofacial clefts showed percentages of 22.2 and 26.8%, among young people aged 12 to 17, and from 15 to 19 years old, respectively.^12,24^ Still on the national scene, in another study in which 15-year-olds participated, 67% said they used alcoholic beverages, with 87.5% at low-risk consumption, and 12.5% considered at risk.^18^ Internationally, the percentages were 26.5% in the Dominican Republic, and 60.6% in Mexico.^25,26^

Although adolescents with orofacial clefts present psychosocial vulnerability, their experience and repercussions depend on various contexts, including established situational coping modalities, such as religiosity. In fact, among the participants in the present study, especially those who did not use alcohol or with low-risk alcohol use, there were high levels of religiosity in all dimensions. Other investigations carried out with adolescents with orofacial clefts showed similar results.^7,8^

On the other hand, among adolescents with risky or harmful alcohol use, lower scores were observed in all dimensions of religiosity, especially NOR. It is noteworthy that OR can exert an important influence on lifestyle habits, as it is made up of statutes, dogmas, norms, rules and conducts, which prohibit and/or condemn, among others, the use of legal and illicit drugs.^27,28^

Thus, investment in religious spaces, including places of coexistence and maintenance of bonds, can result in greater adherence to different types of cults, encouraging religious institutions as a source of support.^27^

Although the percentage of adolescents with risky or harmful alcohol use appears to be small, identifying possible strategies to mitigate this situation is essential, as complete eradication is challenging. It should be noted that alcohol is recognized as the psychoactive substance most used by adolescents, whose harmful effects are physical and mental, in the short and long term, being also associated with numerous chronic and infectious diseases.^9,10,11,13^

In addition, risky or harmful alcohol use was significantly more frequent among older male adolescents who had a religion but did not practice it. In fact, protection from alcohol use is strongly related to sociodemographic variables.^29^

In relation to gender, different investigations showed a predominance of alcohol use among male adolescents, associated with cultural and socialization issues.^14,26^ In another study, religious norms unfavorable to alcohol consumption protected against risky consumption among exclusively heterosexual women, but not among sexual minorities.^15^

Although most adolescents in this study reported having a religion, some declared that they did not belong to a religious denomination or practiced any religion. These findings may reflect weakened bonds between adolescents and aspects of religiosity that are sometimes influenced by normative pressures from family members and society. Indeed, adolescents can replicate values absorbed under the influence of their parents’ spiritual and religious contexts, although they have not incorporated or do not experience them widely. As a result, they tend to partially enjoy its benefits.^7,8^ Furthermore, it must be considered that religiosity involves broad and dynamic concepts that, in turn, can influence perceptions and meanings.^29^

In the present study, risky or harmful alcohol consumption was more prevalent among adolescents without a religion or who did not practice it. Having a religion represented a significant subset of the social network, where experiences, including healthy lifestyle habits, are shared, in addition to directly influencing its members.^17^

In summary, the variables associated with alcohol use are multifaceted and complex, requiring monitoring thereof by public authorities, especially the health system, in addition to planning and implementing interventions to minimize this problem.

Furthermore, among the adolescentes in this study, the lower the levels of both NOR and IR, the higher the prevalence of risky or harmful alcohol use. This finding corroborates the results of a recent systematic review which concluded that both, religiosity and spirituality exert an important protective factor for adolescents against alcohol use.^29^ Similar results were evidenced in other investigations.^15,16^ Briefly, protection of religiosity refers to postponing contact with alcohol, minimizing its consumption and, consequently, its harmful effects.^18,25^

In addition to the protective mechanisms of religiosity not being fully elucidated and those already described previously, it is believed that they are related to the fact that social networks associated with the religious environment are more receptive to messages regarding healthy habits, which include the prohibition of legal and illegal drugs, and the role of the family and friends as important sources of support for non-consumption of alcohol, guided by influence and social interaction.^9,10,11,25^

Moreover, accessing approaching and attending temples, including churches and synagogues, practicing individual and collective prayers, meditation, social support from members of the same religious denomination, having a religious identity and connecting with the transcendent, are established as strengthening strategies and resilience, which certainly promote healthy lifestyle habits, which include abstaining from alcohol use.^14,30^ However, additional research is needed to elucidate how, in fact, religiosity acts as protection.

Finally, it is pertinent to point out some limitations, such as the fact that, although the institution, the setting for this research, has a national scope, due to the availability of centers in different locations, the population attended has been concentrated in the southeast region; thus, the results may not represent the national reality, especially when considering the country’s continental dimensions, as well as the rich regional cultural and socio-religious symbolic apparatus. Therefore, further investigations are necessary to elucidate such differences.

Additionally, it should be considered that some adolescents may have had difficulties in admitting the use of alcohol, either due to retaliation from their parents or restrictions linked to religion, which may have influenced, in some way, the results, especially when considering hybrid data collection.

However, the contributions of this investigation are evident, reinforcing the hypothesis that religiosity, in most of its dimensions, helped attenuate alcohol use among adolescents with orofacial clefts. In this sense, the great challenge consists in transforming these findings into preventive and therapeutic interventions, by using, for example, the religiosity variables as indicators of health and well-being, in addition to its viability, to identify adolescents at increased risk of using alcohol and possible inclusion in prevention and rehabilitation programs. Another significant benefit refers to its novelty, as it addresses adolescents with the most prevalent facial malformation in the population.

In general, adolescents with orofacial clefts showed high levels of religiosity. Confirming the hypothesis of this study, those with higher levels of organizational and intrinsic religiosity used less alcohol. On the other hand, males, older adolescents, and non-practitioners of religion were associated with increased odds of risky or harmful alcohol use.

In a nutshell, these findings reinforce the presumption that religiosity, in most of its dimensions, mitigated the use of alcohol among adolescents with orofacial clefts, besides strengthening its use as an indicator of health and well-being.

## Data Availability

The database that originated the article is available with the corresponding author.
